# Modulating the vascular behavior of metastatic breast cancer cells by curcumin treatment

**DOI:** 10.3389/fonc.2012.00161

**Published:** 2012-11-15

**Authors:** Anna L. Palange, Daniele Di Mascolo, Jaykrishna Singh, Maria S. De Franceschi, Claudio Carallo, Agostino Gnasso, Paolo Decuzzi

**Affiliations:** ^1^Department of Translational Imaging, The Methodist Hospital Research Institute, HoustonTX, USA; ^2^Department of Nanomedicine, The Methodist Hospital Research Institute, HoustonTX, USA; ^3^Department of Experimental and Clinical Medicine, University of Magna GraeciaCatanzaro, Italy

**Keywords:** circulating tumor cells, vascular adhesion, parallel plate flow chamber, curcumin treatment, metastasis

## Abstract

The spreading of tumor cells to secondary sites (*tumor metastasis*) is a complex process that involves multiple, sequential steps. Vascular adhesion and extravasation of circulating tumor cells (CTCs) is one, critical step. Curcumin, a natural compound extracted from *Curcuma longa*, is known to have anti-tumoral, anti-proliferative, anti-inflammatory properties and affect the expression of cell adhesion molecules, mostly by targeting the NF-κB transcription factor. Here, upon treatment with curcumin, the vascular behavior of three different estrogen receptor negative (ER^–^) breast adenocarcinoma cell lines (SK-BR-3, MDA-MB-231, MDA-MB-468) is analyzed using a microfluidic system. First, the dose response to curcumin is characterized at 24, 48, and 72 h using a XTT assay. For all three cell lines, an IC_50_ larger than 20 µM is observed at 72 h; whereas no significant reduction in cell viability is detected for curcumin concentrations up to 10 µM. Upon 24 h treatment at 10 µM of curcumin, SK-BR3 and MDA-MB-231 cells show a decrease in adhesion propensity of 40% (*p* = 0.02) and 47% (*p* = 0.001), respectively. No significant change is documented for the less metastatic MDA-MB-468 cells. All three treated cell lines show a 20% increase in rolling velocity from 48.3 to 58.7 µm/s in SK-BR-3, from 64.1 to 73.77 µm/s in MDA-MB-231, and from 57.5 to 74.4 µm/s in MDA-MB-468. Collectively, these results suggest that mild curcumin treatments could limit the metastatic potential of these adenocarcinoma cell lines, possibly by altering the expression of adhesion molecules, and the organization and stiffness of the cell cytoskeleton. Future studies will elucidate the biophysical mechanisms regulating this curcumin-induced behavior and further explore the clinical relevance of these findings.

## INTRODUCTION

The spreading of primary tumors to secondary sites (*tumor metastasis*) is responsible for a dramatic decrease in survival rate, with about 90% of the deaths in cancer patients being related to metastasis (Wittekind and Neid, 2005). Although, for a long time, this has been considered as a late stage process in tumor growth, recently it has been realized that metastatic niches can form and progress almost simultaneously with the primary mass (Klein, 2009). Metastasis is a multi-step process where tumor cells have to overcome several barriers before growing at secondary sites: (i) invade the normal tissue surrounding the tumor mass; (ii) enter the bloodstream; (iii) survive in the circulatory system; (iv) leave the blood stream and infiltrate the normal tissue; and (v) proliferate there, evading the immune system surveillance (Morris et al., 1997; Fidler, 2003; Chaffer and Weinberg, 2011). Each of these steps represents an impediment to the distant spreading of the disease that only a few primary tumor cells (one CTC out of a billion of blood cells) can overcome. Yet, metastasis occurs very often in cancer patients (Wong et al., 2001; Chambers et al., 2002).

A critical step in this process is the vascular transport of tumor cells in the hostile hemodynamic environment and the extravasation to secondary sites (Wyckoff et al., 2000). Circulating tumor cells (CTCs), transported by the blood flow, are subjected to *mechanical stress*, generated by high shear forces and collisions with other cells; and *immunological stress*, as these cells can be recognized and attacked by lymphocytes (Al-Mehdi et al., 2000; Chang et al., 2008; Vivier et al., 2008). Extravasation and infiltration are generally associated with pro-metastasis modifications of the CTCs (i.e., epithelial–mesenchymal transition) that originate in the primary mass and continue in the circulation (Iwatsuki et al., 2010; Labelle et al., 2011). The actual mechanism for extravasation depends on the tumor type and metastatic environment and can involve the (i) geometrical trapping of CTCs in narrow blood vessels (diameter <40 µm); (ii) adhesion to endothelial cells, in a leukocyte-like fashion, mediated or not mediated by platelets; and (iii) a combination of these mechanisms (Ribatti et al., 2006; Miles et al., 2008).

**FIGURE 1:**
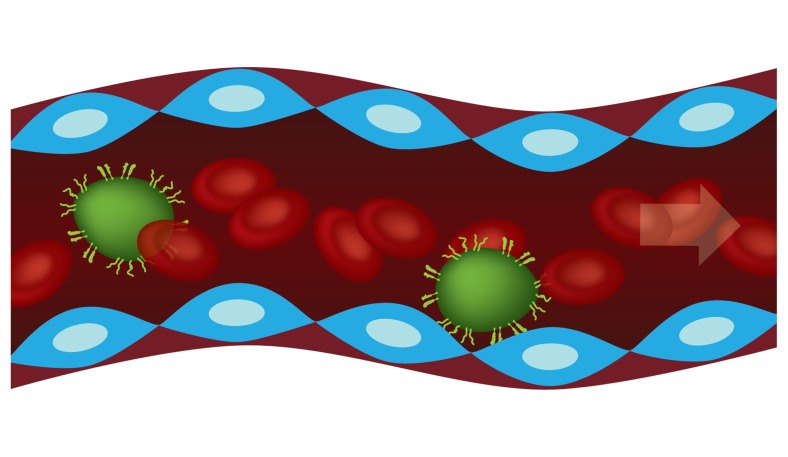
**Schematic representation of circulating tumor cells (CTCs in green) transported by the blood flow and tethering the endothelial cells (blue) through ligand molecules**.

In this study, the main focus is on the vascular adhesion of CTCs under flow, as schematically shown in **Figure 1**. This process consists of two sequential steps: (i) CTC rolling over the vascular walls, regulated by the engagement of endothelial E-selectin and P-selectin with tumor cell glycoprotein such as CD44, CEA, CD24, sulfate glycosaminoglycans (CS-GAGs), sialylated glycosphingolipids; (ii) CTC firm arrest, regulated by other endothelial receptors (ICAM-1, VCAM-1), and different families of integrin molecules (α_v_β_3,_ β_2_ integrins; Wirtz et al., 2011; Geng et al., 2012). In organs with high occurrence of metastasis, such as the liver, this picture is even more complicated in that the characteristic discontinuous and fenestrated endothelium leaves the underlying extracellular matrix directly accessible to CTCs. Moreover, vascular adhesion is also supported by the reorganization and deformation under flow of the cell cytoskeleton. Although tumor cells do not exhibit a leukocyte-like cortical cytoskeleton which is capable of extensive and rapid deformation, it is possible that CTCs exposed to shear stresses in the circulation could undergo transformations facilitating attachment to the vessel walls (Davies et al., 2005). Indeed, firm arrest is a necessary condition for the subsequent extravasation and colonization of the surrounding tissue.

The development of therapeutic agents against metastatic disease is still in its infancy, due to a lack in understanding the leading pathways and, most importantly, their alterations in secondary tumor cells. Also, most of the efforts have been traditionally oriented to eradicate tumor cells already proliferating at the secondary sites, neglecting the opportunity of blocking or modulating one or multiple steps in the metastatic cascade (Chambers et al., 2000). Novel micro- and nanotechnologies detecting, capturing, and characterizing CTCs are providing new and more accurate information on the biomechanical properties of malignant cells in the circulation. Microfluidic systems used as diagnostic tools are demonstrating the significant correlation between higher CTC counts in blood and lower patient survival (Cristofanilli et al., 2004; King et al., 2009; Han et al., 2010; Hughes and King, 2012). It is then reasonable to speculate that novel therapies, possibly nanoparticle-based, could open new avenues for an effective treatment of metastatic diseases by eradicating, or dramatically lowering, the number of CTCs.

In this work, the effect of curcumin on the rolling and adhesion mechanics under flow of three estrogen receptor (ER^–^) breast adenocarcinoma cell lines, namely SK-BR-3, MDA-MB-231, and MDA-MB-468, is analyzed. These cell lines were chosen for their different metastatic potential. Curcumin is a natural multi-target compound with anti-tumoral and anti-inflammatory properties (Kumar et al., 1998; Ray et al., 2003; Kunnumakkara et al., 2008; Binion et al., 2009; Yodkeeree et al., 2010). Their rolling velocity and adhesion propensity are measured experimentally upon treatment with curcumin, using a parallel plate flow chamber system.

## MATERIALS AND METHODS

### MATERIALS AND CHEMICALS

SK-BR-3, MDA-MB-231, MDA-MB-468 breast adenocarcinoma cells were obtained from the American Type Cell Culture Collection (ATCC) and cultured in the recommended medium. SK-BR-3 were grown at 37°C, under a humidified 5% CO_2_ and 95% air at one atmosphere, MDA-MB-231 and MDA-MB-468 were grown at 37°C in a free gas exchange with atmospheric air. Collagen type I from calf skin was obtained from Sigma Aldrich (St. Louis, MO, USA). Curcumin (diferuloylmethane) and dimethyl sulfoxide (DMSO) were obtained from Fisher Scientific. XTT kit was obtained from Trevigen (Gaithersburg, Maryland).

### CYTOTOXIC EFFECT OF CURCUMIN AND XTT ASSAYS

Curcumin was first dissolved in DMSO as a 10 mM stock solution and subsequently diluted in cell culture medium. Medium containing the same amount of DMSO was used as control at concentrations not exceeding the 0.1% v/v of the culture medium. Cells were incubated with curcumin at 1, 10, 20, and 40 µM, at three time points namely 24, 48, and 72 h. Cell proliferation was measured with conventional XTT reduction assays. Briefly, SK-BR-3, MDA-MB-231, and MDA-MB-468 cells were inoculated at a density of 5 × 10^3^ cells in 96-well plates for 24 h in 200 µl of recommended medium. The culture supernatant was then removed and medium containing the above mentioned curcumin concentrations was added to cells, subsequently incubated for 24, 48, and 72 h. After that, XTT-dye was mixed with phenol-free medium and added to the samples. The plate was incubated for at least 1 h before reading. The absorbance of XTT-formazan dye was then measured using a microplate reader at 490 nm. Twenty-five repetitions were performed for each time point and concentration.

### PARALLEL PLATE FLOW CHAMBER FOR CELL ROLLING AND ADHESION

Before flow adhesion experiments, cells were first incubated with medium containing no FBS for 7 h and then treated with or without curcumin at 10 µM for 24 h. Subsequently, cells were detached from culture dishes by mild trypsinization (0.25% trypsin/EDTA) for 2 min at 37°C and incubated at 37°C for 1 h to allow the regeneration of surface glycoproteins. After that, cells were washed in PBS and resuspended at 10^6^ cells/ml in serum-free medium containing 0.1% bovine serum albumin, following standard protocols (McCarty et al., 2000).

**FIGURE 2:**
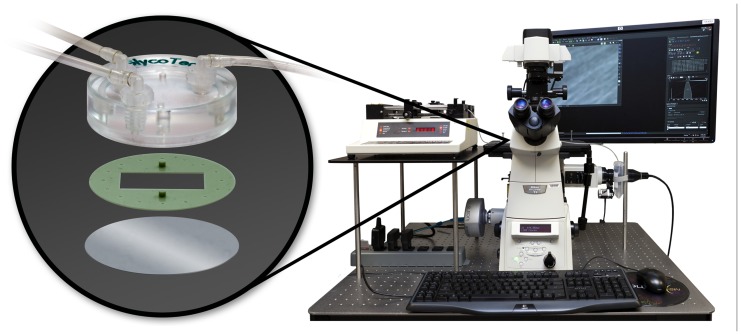
** The parallel plate flow chamber system composed of the microfluidic chamber (deck, gasket, and microscope slide represented in the oval at left); the syringe pump, the epifluorescent microscope and a desktop computer**.

The rolling and adhesion behavior of the tumor cells, was studied using a parallel plate flow chamber system (Chen et al., 1997; Brown and Larson, 2001; GlycoTech Corporation; **Figure 2**). The system comprises a commercially available flow chamber, a syringe pump (Harvard Apparatus, MA), an inverted epifluorescent microscope (Nikon Ti-Eclipse) and a desktop computer for data storage and analysis. The main constituents of the flow chamber are a deck, a rubber gasket and a glass slide. The rubber gasket defines the geometry of the flow region (length *l *= 20 mm; width *b *= 10 mm; height *h *= 0.254 mm). The coverslips (slide), closing the bottom of the chamber, were covered with a uniform collagen layer. In particular, autoclaved 35 mm coverslips were covered by a collagen solution obtained diluting collagen type I from calf skin (Sigma Aldrich) in PBS to reach a concentration of about 50 mg/cm^2^. After about 5 h at room temperature, the cover slips were rinsed in PBS and left under the bio-hood to dry. To perfuse the solution at a fixed wall shear rate *S*, flow rate *Q *is finely controlled through the syringe pump given that *S* = 6*Q/bh*^2^.

After assembling all the components, the system was placed on the stage of an epifluorescent microscope (Nikon Ti-Eclipse). The Andor’s Luca EM S camera utilizes a 658 × 496 “interline frame transfer” EMCCD sensor to acquire the region of interest (ROI) and allows for real time monitoring. For each experiment 10^6^/ml cells were perfused in 1 ml of serum-free medium, at a wall shear rate of *S* = 10 s^–^^1^ (mimicking the circulation environment of microvascular tumor vessels). Eight experiments were performed for the SK-BR-3 and MDA-MB-468-treated and untreated cells, and 10 experiments for the MDA-MB-231. All the experiments lasted 12 min each.

Through offline data analysis on the movies derived from each experiment, the number of firmly adhering cells on the substrate and their mean rolling velocity were quantified. Firmly adhering cells were those cells staying within the region of interest (10× objective, ROI = 658 × 496 pixel) till the end of the experiment. This number was normalized by the total number of injected cells and the area of the ROI (~0.33 × 10^–^^6^ m^2^) deriving the adhesion propensity. The rolling velocity was calculated as the displacement of the centroid of the cells divided by the time interval of their observation (on average about 10 s). The rolling velocity was calculated for 12 cells in three different SK-BR-3 experiments (*n* = 36), and six cells in eight different MDA-MB-231 and MDA-MB-468 experiments (*n* = 48), for both the treated and the control group.

### STATISTICAL ANALYSIS

Data are expressed as means ± SD. Statistical significance of differences between means was determined by one-way ANOVA. Probability values of *p* < 0.05 were considered statistically significant.

## RESULTS AND DISCUSSION

**FIGURE 3:**
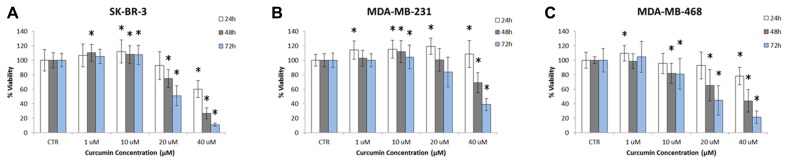
**Proliferation of (A) SK-BR-3, **(B)** MDA-MB-231, **(C)** MDA-MB-468 cells exposed to different concentrations of curcumin (1, 10, 20, and 40 µM) at three time points (24, 48, and 72 h)**. Note that cell proliferation is not affected at curcumin concentrations below 10 µM up to 72 h (*n* = 25). The asterisk symbol “*” denotes significant difference (*p* < 0.05) as compared to control (Ctr).

The cytotoxic effect of curcumin on SK-BR-3, MDA-MB-231, and MDA-MB-468 cells was analyzed using a XTT proliferation assay. Cells were incubated with curcumin at different doses, namely 1, 10, 20, and 40 µM, and for three time points, namely 24, 48, and 72 h. The cell viability was measured following the protocols described in Section “Materials and Methods” and is reported in the bar charts of **Figure 3**, for 25 repetitions in each cell line. As expected the percentage of viable cells reduces as the concentration and the duration of the treatment increase. Interestingly, for concentrations lower and equal to 10 µM, curcumin has no significant effect on the cell viability. Note that for the control experiments, cell culture medium was added with the same amount of DMSO used in the actual experiments for dissolving curcumin. This volume was about 0.1% of the total medium volume and no sign of toxicity was observed on the cells. For larger concentrations, 20 and 40 µM, the curcumin treatment limits cell proliferation in a dose and time dependent manner. Cell viabilities lower than 50% can only be observed at the highest concentration and longer time points (48 and 72 h). The IC_50_ is reached at ~20 µM for the SK-BR-3 and MDA-MB-468 at 72 h; and at ~40 µM for the MDA-MB-231 at 72 h. This is in agreement with most literature on curcumin (Aoki et al., 2007). From this assay, the exposure to 10 µM of curcumin for 24 h was considered a mild treatment inducing no significant, direct effects on cell viability.

**FIGURE 4:**
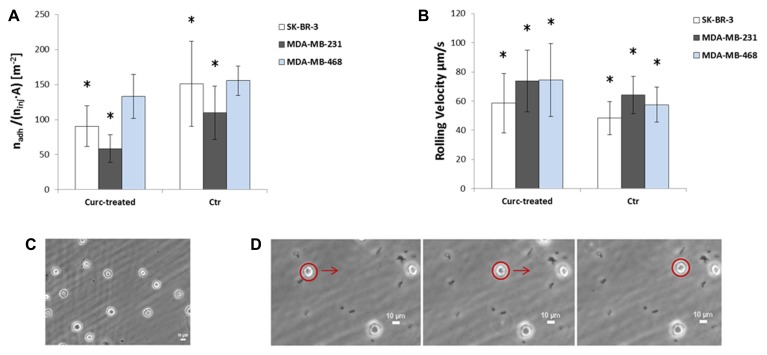
**(A)** Adhesion and **(B)** rolling behavior of SK-BR-3 (white), MDA-MB-231(gray), MDA-MB-468 (blue) cells treated with curcumin (Curc-treated) at 10 µM for 24 h, and not treated cells (Ctr). The number of firmly adhering cells (*n*_adh_) is normalized by the total number of injected cells (*n*_inj_ = 10^6^) and the area of the region of interest (*A* = 0.33 × 10^–^^6^ m^2^). Data are plotted as mean ± SD. The asterisk symbol “*” denotes significant difference (*p* < 0.05) as compared to control (Ctr). **(C)** Image of firmly adhering SK-BR3 cells on the substrate at the end of the experiment. **(D)** Captured frames showing the displacement of a rolling cell on the substrate.

The effect of curcumin on the rolling and adhesion behavior of the three breast adenocarcinoma cells lines SK-BR-3, MDA-MB-231, and MDA-MB-468 was analyzed using a parallel plate flow chamber, traditionally employed for the analysis of leucocyte adhesion and rolling (Lawrence et al., 1987). The cells, mildly pretreated with curcumin (24 h at 10 µM) as detailed in Section “Materials and Methods,” were infused in the apparatus depicted in **Figure 2** and their rolling velocity and firm adhesion was quantified via post processing of the images taken with an epifluorescent microscope. The bar charts of **Figure 4** present the results of the flow chamber assay. **Figure 4A** shows the adhesion propensity of the curcumin-treated cells (Curc-treated) and untreated cells (Ctr), as the ratio between the absolute number of adhering cells (*n*_adh_), the total number of injected cells (*n*_inj_ = 10^6^), and the area of the region of interest (*A* = 0.33 × 10^–^^6^ m^2^). SK-BR-3, MDA-MB-231, and MDA-MB-468-treated cells present an adhesion propensity of 90.7 ± 29.2, 58.58 ± 19.70, and 132.97 ± 31.34#/m^2^, respectively. This means that for a vessel of 50 µm in diameter with a 500 µm length (vascular area ~1 mm^2^), about 300 SK-BR-3, 180 MDA-MB-231, and 400 MDA-MB-468 cells would firmly adhere under the same biophysical conditions and assuming that 10^6^ CTCs enter the specific vessel. On the other hand, the untreated cells exhibit a larger adhesion propensity of 151.1 ± 60.84, 109.69 ± 38.19, and 155.42 ± 20.74#/m^2^^,^ for the SK-BR-3, MDA-MB-231, and MDA-MB-468, respectively. The difference between the two populations (Curc-treated and Ctr) is significant for the SK-BR-3 and even more for the highly metastatic MDA-MB-231 cells. More specifically, the curcumin-treated SK-BR-3 cells show a 40% decrease (*p* = 0.02) in adhesion and the curcumin-treated MDA-MD-231 a 47% decrease (*p* = 0.001) as compared to the control group. Conversely, the difference in adhesion propensity between curcumin-treated MDA-MB-468 cells and the control group is not statistically significant (*p* = 0.099).

Rolling velocity is reported in **Figure 4B**. This physical quantity was estimated as the ratio between the displacement of the cell centroid over the corresponding observation time. Despite the large standard deviation, this analysis shows that there is a significant difference between the two groups (Curc-treated and Ctr) in all three cell lines: SK-BR-3 and MDA-MB-231-treated tumor cells roll ~1.2 times faster than the control cells (*p* = 0.01, *p* = 0.009, respectively), MDA-MB-468-treated tumor cells rolls ~1.3 times faster than the control cells (*p* = 0.00005). In particular, the rolling velocities are 58.66 ± 20.29 and 48.33 ± 11.3 µm/s for SK-BR-3-treated and untreated cells, 73.77 ± 21.21 and 64.1 ± 12.89 for MDA-MB-231-treated and untreated cells, 74.38 ± 24.88 and 57.49 ± 11.96 for MDA-MB-468-treated and untreated cells, respectively. Indeed, the higher rolling velocity correlates well with the lower adhesion propensity observed above.

These preliminary results collectively would suggest that mild treatments with curcumin could impair cell adhesion and increase cell rolling under flow over normal, untreated cells. Indeed, this could reduce the metastatic potential of CTCs. Understanding the mechanisms regulating the observed alteration in the behavior of SK-BR-3, MDA-MB-231, and MDA-MB-468 cells is out of the scope of this preliminary study. However, it has already been reported that curcumin treatments alter the organization of microfilaments and increase the overall quantity of F-actin. This would affect cell motility and deformability, which are crucial elements in supporting tumor cell circulation and survival in the blood stream. Moreover, recently it has been shown that CTCs reattach in distant tissues by a mechanism that is tubulin-dependent and suppressed by polymerized actin (Holy, 2004; Matrone et al., 2010). In addition, curcumin is known to decrease the expression and modulate the activity of membrane adhesion molecules by acting on the transcription factor NF-κB. For instance, the curcumin inhibition of NF-κB completely blocked TNF-α induced expression of adhesion molecules (ICAM-1, VCAM-1, and E-selectin) on HUVECs and human intestinal microvascular endothelial cells attenuating leucocytes adhesion (Kumar et al., 1998; Ray et al., 2003; Binion et al., 2009). Therefore, in the present case, it is reasonable to speculate that curcumin effects could depend, at least in part, on a reduced expression or more likely, on the modulation of integrins receptor activity (mostly α_1_β_1_ and α_2_β_1_) thus limiting the adhesion propensity under flow (Park et al., 2006; Ivascu and Kubbies, 2007). Here, it is important to note that integrins, expressed on cellular membrane, can specifically bind to the collagen type I, deposited on the flow chamber glass slide. Also, in organs with high metastatic occurrence, such as the liver, the discontinuous, fenestrated endothelium allows the CTCs to directly interact and bind to ECM components. For this reason, collagen type I, has been also used in flow chamber experiments to assess the adhesive behavior of cells and CTCs (Haier et al., 1999; Wendel et al., 2012).

Finally, curcumin, a natural compound extracted from *Curcuma longa*, has been demonstrated to have a wide spectrum of biological and pharmacological activities. In particular, it exhibits antiviral, antibacterial, antioxidant, anti-inflammatory, anti-proliferative, and anti-angiogenic properties (Aggarwal et al., 2003; Holt et al., 2005). Animal and human studies have suggested its potential use in the treatment of inflammation and cancer, mostly because of its potent effect on the NF-κB pathway (Kawamori et al., 1999; Aggarwal, 2004). However, the major drawback in its clinical use is the very low bioavailability and biodistribution, mostly due to its poor absorption from the gut, rapid metabolism and elimination (Shoba et al., 1998; Anand et al., 2007). The formulation of curcumin into nanoparticles could avoid the drawbacks listed above and enhance its curative properties.

## CONCLUSION

The ER negative breast metastatic cells, SK-BR-3, MDA-MB-231, and MDA-MB-468, cells were treated with curcumin, at three different time points. For sufficiently large curcumin doses (≥ 20 µM), significant cell death is induced at 72 h. Conversely, a mild treatment with curcumin (≤ 10 µM at 24 h), did not show any significant change in cell viability but did affect the vascular behavior of the cells. This was demonstrated by assessing the cell adhesion and rolling velocity in a parallel plate flow chamber system. The SK-BR-3 cells showed a 40% decrease (*p* = 0.02) in cell adhesion propensity and 20% increase (*p* = 0.001) in rolling velocity. The MDA-MB-231-treated cells showed almost a 50% decrease (*p* = 0.001) in cell adhesion propensity and about 15% increase (*p* = 0.009) in rolling velocity. The MDA-MB-468-treated cells did not show any statistically significant decrease in adhesion propensity, but did roll 1.3 times faster than the control group (*p* = 0.00005).

Collectively, these results suggest that mild curcumin treatments of CTCs could lower or even prevent the occurrence of metastasis, by reducing CTCs adhesion at secondary vascular sites. Future works will have to elucidate the mechanisms regulating the observed alteration in tumor cell behavior and the specific pathways involved in each cell line studied, by characterizing the expression and activity of cell membrane receptors, the organization of the cell cytoskeleton and its deformability. However, the proper delivery of sufficient doses of curcumin to CTCs could provide a new strategy to prevent the metastatic spread.

## Conflict of Interest Statement

The authors declare that the research was conducted in the absence of any commercial or financial relationships that could be construed as a potential conflict of interest.
